# Outcomes of anterior component separation versus posterior component separation with transversus abdominis muscle release for large incisional hernias: a systematic review and meta-analysis

**DOI:** 10.1007/s10029-025-03487-5

**Published:** 2025-10-09

**Authors:** Ali Yasen Mohamedahmed, Mohamed Talaat Issa, Shafquat Zaman, Safeya Mohammed, Marwa Yassin Mohamedahmed, Mohammed Hamid, AK Ali Muhammed, Stephen Odogwu, Syed Adnan Kabir

**Affiliations:** 1 Department of General and Hepatobiliary Surgery, University Hospitals of Coventry and Warwick, Coventry, UK; 2 Department of General Surgery, Walsall Manor Hospital, Walsall, UK; 3 College of Medical and Dental Science, School of Medicine, University of Birmingham, Birmingham, UK; 4 Department of General and Colorectal Surgery, University Hospitals of Derby and Burton NHS Foundation Trust, Burton on Trent, Staffordshire, UK; 5 Department of General Surgery, Ibrahim Malik Teaching Hospital, Khartoum, Sudan; 6 Department of General Surgery, Atbara Teaching Hospital, Atbara, Sudan; 7 Department of General Surgery, St Mark’s Hospital, London, UK

**Keywords:** Component separation, Incisional hernia, Meta-analysis, Systematic review, TAR release

## Abstract

**Background:**

Large incisional hernias (IHs), especially with loss of domain, pose significant challenges for repair. Component separation, as a method of repair, allows for adequate coverage of large hernial defects. We compared outcomes of anterior component separation (ACS) versus posterior component separation with transversus abdominis muscle release (PCSTAR) in the repair of large IHs.

**Methods:**

A systematic search of various electronic databases was conducted to identify studies (published between January 1990 - June 2025) comparing ACS and PCSTAR performed for IH repair. The included studies were assessed for risk of bias (RoB) using validated tools appropriate to study design (Cochrane RoB for randomised controlled trials (RCTs), MINORS for non-randomised studies). Our evaluated outcome measures included overall wound complications, surgical site infections (SSI), hematoma and seroma formation, total operative time, length of hospital stay (LOS), and recurrence rate. Data were analysed using RevMan 5.3, employing a random-effects model.

**Results:**

A total of eight studies (three RCTs and five observational studies) with 2293 patients (1573 with ACS and 720 with PCSTAR) were included. The PCSTAR group demonstrated a lower rate of overall wound complications (odds ratio [OR] 2.58, *P* = 0.004) and SSIs (OR 1.72, *P* = 0.05) compared with the ACS group. No significant differences were observed for hematoma (OR 0.87, *P* = 0.51) or seroma formation (OR 1.77, *P* = 0.11), recurrence rate (OR 1.81, *P* = 0.31), operative time (mean difference [MD] -6.57, *P* = 0.77), or LOS (MD -0.67, *P* = 0.16) between the two groups. Overall, RCTs demonstrated a low risk of bias in most domains, whilst non-randomised studies showed moderate methodological quality.

**Conclusion:**

Both component separation techniques demonstrated comparable outcomes and efficacy in the repair of large incisional ventral hernias (IVHs). However, PCSTAR seems to be associated with reduced overall wound complications and SSI rates. A small number of included RCTs mandate that further adequately powered, well-designed RCTs are required to validate these findings.

**Supplementary Information:**

The online version contains supplementary material available at 10.1007/s10029-025-03487-5.

## Introduction

Ventral hernias (VH) pose separate and unique challenges for both patient and surgeons alike, with an estimated 25,000 to 350,000 repaired annually in the UK and USA, respectively [1]. They develop either as primary hernias or secondary to previous surgery (incisional hernia (IH)). Incisional ventral hernias (IVHs) most commonly occur following laparotomies with an incidence of 40% [2].

In the UK, laparotomies performed in the emergency setting vary between an estimated 25,000 and 30,000 cases per annum, translating into a proportion of patients at risk of developing future IVHs [3]. Patients with these types of hernias are often categorised into groups based on associated comorbidities. These include patients with malignant conditions presenting with complications such as bowel obstruction or perforation requiring laparotomies and resection, subsequently being complicated by IHs at sites of tumour extraction. A second group are trauma presentations or benign pathologies leading to gastrointestinal obstruction and perforation. In both sets of patients, prolonged and protracted recovery periods may lead to delays in IH repair. The interim increase in size of the abdominal wall defect can make any repair more difficult.

The component separation technique (CST) developed and popularised in the early 1990 s allows large abdominal wall defects to be repaired without the need for bridging mesh [4]. It achieves this through the medial advancement of abdominal wall muscles via the release of surrounding tissue planes, favouring fascial closure [5]. In anterior component separation (ACS), dissection is performed in the subcutaneous plane, and the external oblique aponeurosis incised lateral to the rectus sheath facilitating closure of the hernial defect [6]. In posterior component separation with transversus abdominis release (PCSTAR) a more recent modification - the retro-rectus space is developed and the transversus abdominis muscle released at its insertion. This creates a large, well-vascularised space for mesh placement, often avoiding the need for subcutaneous dissection. The technique allows closure of large midline defects with reduced risk of wound morbidity compared with ACS, especially in obese and multi-morbid patients [7].

A previously published meta-analysis compared ACS and PCSTAR in terms of wound complications and recurrence rates; however, considerable debate remains within the surgical community regarding the optimal technique [8]. We performed an updated meta-analysis, incorporating the latest published literature, to compare outcomes between ACS and PCSTAR.

## Methods

### Study design

The systematic review and meta-analysis were designed and conducted in accordance with the Cochrane Handbook for Systematic Reviews of Interventions and followed the PRISMA (Preferred Reporting Items for Systematic Reviews and Meta-Analyses) guidelines for reporting [9, 10].

### Data sources and search strategy

A comprehensive search was conducted using various electronic databases and clinical trial registries, including PubMed, Scopus, Cochrane Central Register of Controlled Trials (CENTRAL), Google Scholar, ClinicalTrials.gov, ScienceDirect, and the Virtual Health Library (VHL). The search was performed by two independent reviewers. It used the following terms: ‘component separation’ AND (‘anterior component separation’ OR ‘posterior component separation’ OR ‘TAR release’) AND ‘transversus abdominis’ OR ‘TAR’ AND ‘ventral incisional hernia’ AND ‘midline hernia’. Additionally, the reference list of relevant studies was manually reviewed and interrogated to identify further published studies.

### Study selection

Titles, abstracts, and full texts of the selected studies from the search were independently screened and scrutinised by two reviewers to assess their eligibility against our inclusion criteria. Studies comparing ACS with PCSTAR and published between January 1990 - June 2025 were included. Single-arm trials, case series, case reports, posters, letters to the editor, and review articles were excluded.

Studies reporting outcomes of the surgical techniques of interest (ACS with PCSTAR) were considered eligible for inclusion even if they included a mixed patient population (e.g., primary and recurrent hernias, or ventral and flank hernias). In such instances, inclusion was justified by the relevance of the comparative data reported, and potential heterogeneity was addressed through sensitivity analyses, where possible. Discrepancies between the reviewers regarding the inclusion of studies were resolved through consultation with the wider authorship team. Although the study was not prospectively registered on a systematic review registry such as PROSPERO [11], it was conducted in accordance with PRISMA guidelines and adhered to Cochrane methodology to ensure transparency and quality.

### Data extraction and collection

Two reviewers independently performed data extraction and collected the following information: name and details of the first author, year of study publication, country of origin, study design, total number of participants, patient characteristics/demographics, and study outcomes. Where disagreements arose during this process, a third author from the research team was consulted to reach a consensus.

### Study outcomes

Our primary outcomes were postoperative complications (overall wound morbidity, surgical site infection (SSI), seroma and hematoma formation). SSIs were defined according to the National Healthcare Safety Network (NHSN, CDC 2022) criteria, which categorises SSIs into superficial incisional, deep incisional, and organ/space infections occurring within 30 days of the procedure (or within 90 days if prosthetic material is implanted), and meeting specific clinical, laboratory, or radiological findings [12]. The overall wound morbidity equated to the sum of all wound complications, including SSI, hematoma, and seroma formation.

Our analysed secondary outcomes were: total operative time (minutes), length of hospital stay (LOS), and hernia recurrence rate. Recurrence was measured after complete wound healing and hospital discharge following the index procedure, and diagnosed clinically and/or radiologically.

### Risk of bias assessment

The Cochrane risk of bias tool and the Newcastle-Ottawa Scale (NOS) assessed bias risk in the included RCTs and observational studies [13]. Using the NOS scoring system, studies were categorised as low (score of 9), moderate (scores of 7 or 8), or high risk (scores less than 6). Any disagreements between assessors during this process were resolved by consulting an independent author.

For non-randomised studies, methodological quality was assessed using the Methodological Index for Non-Randomised Studies (MINORS) tool [14]. This validated scoring system includes 12 items, each scored from 0 to 2, with a maximum possible score of 24 for comparative studies. Two reviewers independently scored each study, and any disagreements were resolved through discussion and reaching a consensus.

## Statistical analysis

All statistical analyses were conducted using Review Manager (RevMan) version 5.4, developed by the Nordic Cochrane Centre. For continuous variables, the mean difference (MD) was calculated with a 95% confidence interval (CI). For categorical outcomes, the odds ratio (OR) was determined using the Mantel-Haenszel method. Where both comparison groups reported zero events in 25% of the included studies, the risk difference (RD) was used. If continuous data were presented as median and interquartile range (IQR), the method outlined by Hozo et al. was applied to estimate the corresponding mean and standard deviation (SD) [15].

Statistical significance was defined as a P value of ≤ 0.05, with a 95% CI applied throughout the analysis. A random-effects model was used for all outcome analyses. Heterogeneity among the studies was assessed using the I² statistic and Chi-square (χ²) test, with significant heterogeneity indicated by a *P* ≤ 0.05 or an I² value exceeding 50%.

The potential heterogeneity and reliability of the results were assessed using sensitivity analyses. This included recalculating risk ratios (RR) or RD for categorical outcomes and performing a leave-one-out analysis to evaluate the impact of individual studies.

## Results

**Fig. 1:**
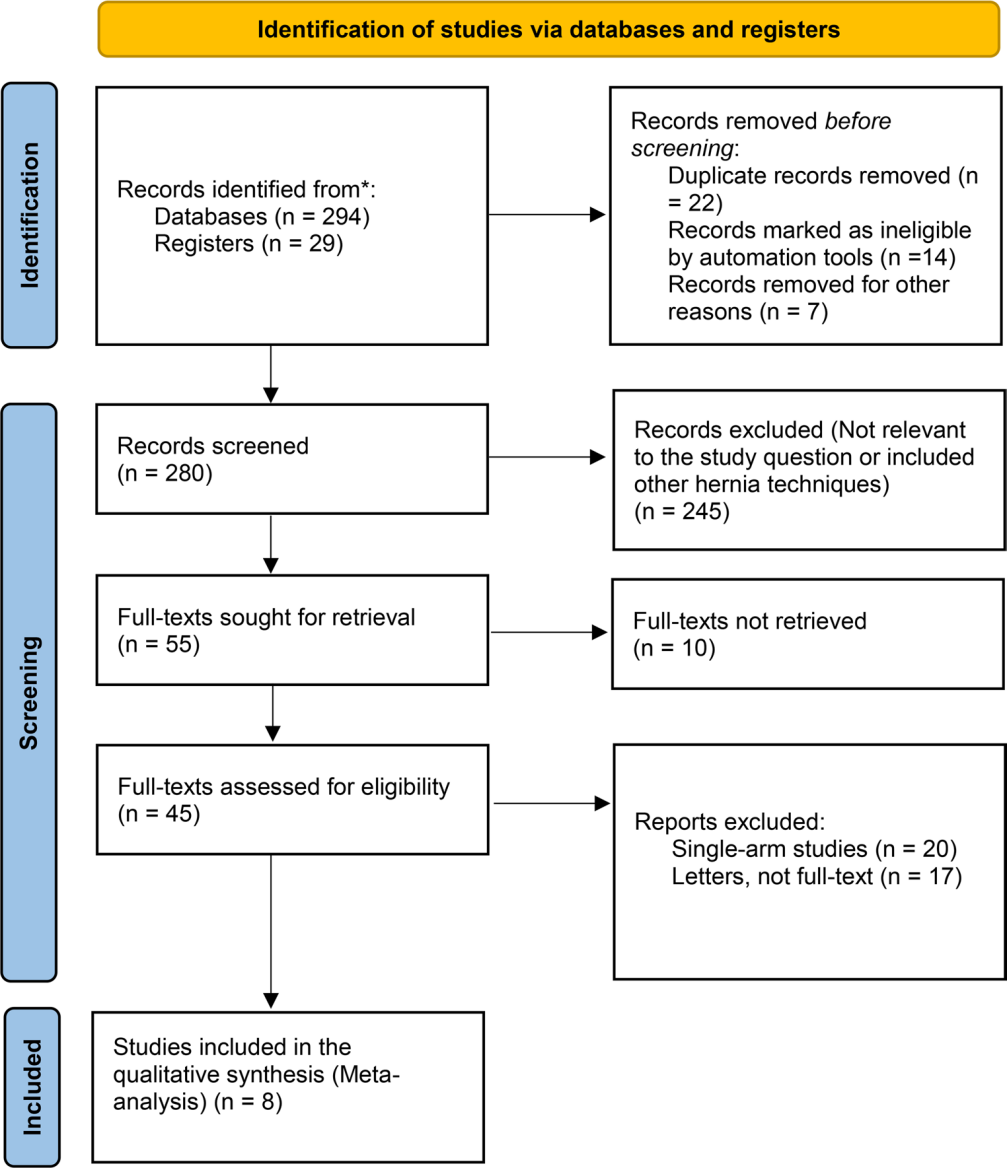
Risk of bias assessment of included randomised controlled trials (RCTs) Overall wound complications

A total of 323 studies were identified through the systematic search of the aforementioned data sources. Screening titles and abstracts and removing duplicates resulted in the exclusion of 280 studies, leaving 45 for further review. Full manuscripts were then assessed against the eligibility criteria, resulting in eight studies [16–23] being included in the meta-analysis. Three studies included in the data synthesis were RCTs [18, 19, 23] and five were non-randomised observational studies [16, 17, 20–22]. The PRISMA flow chart is presented in Fig. 1, and the characteristics of the included studies are summarised in Table 1.

**Table 1:** Baseline characteristics of the included studies. ACS, anterior component separation; PCS, posterior component separation with transversus abdominis muscle release; USA, united States of America; AWR, abdominal wall reconstruction; RCT, randomised controlled trial; BMI, body mass index; HbA1c, glycated haemoglobin; CST, component separation technique; cm, centimetre; CT, computed tomography

Study	Country	Type of Study	Number of patients	Inclusion/Exclusion criteria	Follow-up (months)
Karpata 2012 [16]	USA	Retrospective cohort	ACS: 56 PCS: 55	**Inclusion criteria**: All ACS or PCSTAR operations performed by a single surgeon between 2006 and 2011	ACS: 9.1 (3-50.5) PCSTAR: 6.8(3-49.1)
Blair 2016 [17]	USA	Prospective cohort	ACS: 95 PCS: 33	**Inclusion criteria**: All patients underwent AWR between 2005 to 2015	16.4
Albalkiny 2018 [18]	Egypt	RCT	ACS: 20 PCS: 20	**Inclusion criteria**: Midline incisional hernias with defects larger than 5 cm in width **Exclusion criteria**: Patients with defects width less than 5 cm	12
Bochicchio 2019 [19]	USA	RCT	ACS: 61 PCS: 59	**Inclusion criteria**: Adults ≥ 18 years with ventral hernia ≥ 200 cm2, BMI ≤ 40, HbA1c < 7%, tobacco-free ≥ 6 weeks. **Exclusion criteria**: Moribund patients, life expectancy < 1 year, active infection, collagen disorders, malignancy, ASA 4–6	12
Maloney 2019 [20]	USA	Prospective cohort	ACS: 25 PCS: 88	**Inclusion criteria**: All open AWR utilising CST, including incisional, ventral, and flank hernias	ACS: 21.3 + 22.2 PCSTAR: 20.7 + 23.2
Rodriguez 2021 [21]	Spain	Retrospective cohort	ACS: 1193 PCS: 343	**Inclusion criteria**: Patients undergoing a CST technique (anterior or posterior) between July 2012 and December registered on online database registry	14.7
Toma 2023 [22]	Romania	Retrospective cohort	ACS: 101 PCS: 101	**Inclusion criteria**: adult patients (over 18 years old) with post-operative primary or recurrent, median abdominal wall defects larger than 6 cm and with complete fascial closure and non-contaminated wound **Exclusion criteria**: Emergency operation, lateral and parastomal hernias, patients without a preoperative CT scan, infected wound, anterior fascia could not be closed, and patients with single-sided separation	3
Demetrashvili 2025 [23]	Georgia	RCT	ACS: 22 PCS: 21	**Inclusion criteria**: Adults > 18 yrs with large midline ventral hernia > 150 cm2, complete fascial closure **Exclusion criteria**: Strangulated/lateral/parastomal hernias, inability to close anterior fascia, bowel resection, ASA 4–5, immunocompromised, patient preference/refusal	ACS: 32.1 PCS: 33.4

## Patient characteristics and baseline demographics

A total of 2,293 patients were included in this analysis (1,573 in ACS group and 720 patients in PCSTAR group). The ACS group consisted of 842 males (53.5%) and 731 females (46.5%), whilst the PCSTAR group had 378 males (52.5%) and 342 females (47.5%), demonstrating comparable gender distribution between the two groups. Notably, one study included a proportion of patients with flank hernias in addition to ventral hernias [20], and two studies included both primary and recurrent ventral hernias [20, 22]. The average age of our cohort was 57.5 years (ACS group) and 58.6 years (PCSTAR group), respectively. Mean body mass index (BMI) was also similar across the two groups (32.7 kg/m² for ACS and 32.5 kg/m² for PCSTAR), indicating Class 1 obesity.

For co-morbidities, 18.8% of patients in the ACS group and 36.4% in the PCSTAR group were either current or former smokers. Diabetes mellitus was reported in 21.6% of ACS and 28.2% of PCSTAR patients. COPD was present in 15.3% of ACS and 15.7% of PCSTAR patients, reflecting a similar pulmonary risk profile between the two groups. The demographics and operative profiles of the included patients are summarised in Table 2.

**Table 2:** Demographics of included patients. ACS, anterior component separation; PCS, posterior component separation with transversus abdominis muscle release; BMI, body mass index; SD, standard deviation; n, number of patients; COPD, chronic obstructive pulmonary disease; cm, centimetre; NA, not available

Study	Approach	Age (years) Mean ± SD	Gender (male) *n* (%)	BMI Mean ± SD	Smokers *n* (%)	Diabetics *n* (%)	COPD *n* (%)	Hernia defect size in cm2 Mean ± SD	Size of mesh coverage (cm2) Mean ± SD
Krpata 2012 [16]	ACS	59.6 *±* 11.2	18 (32.1)	38 *±* 10.1	15 (26.8)	37 (66.1)	19 (33.9)	531 *±* 324.3	NA
	PCS	54.7 *±* 11.7	34 (61.8)	32.5 *±* 7	12 (21.8)	19 (34.5)	14 (25.5)	471.5 *±* 324.2	
Blair 2016 [17]	ACS	58 *±* 10.9	56 (58.9)	33.2 *±* 7.8	15 (15.7)	27 (28.4)	NA	338.9 *±* 203.7	795.3 *±* 444.3
	PCS	59.8 *±* 10.5	26 (78.8)	33.1 *±* 6.4	3 (9.1)	7 (21.2)		223.6 *±* 147	919.6 *±* 261
Albalkiny 2018 [18]	ACS	NA	10 (50.0)	35.2 *±* 4.92	9 (45.0)	5 (25.5)	4 (20.0)	10.6 *±* 3.33	NA
	PCS		11 (55.0)	35.55 *±* 3.73	12 (60.0)	4 (20.0)	4 (20.0)	11.1 ± 3.385	
Bochicchio 2019 [19]	ACS	56.3 ± 11.2	20 (66.7)	32.2 ± 5.6	16 (53.3)	6 (20.0)	2 (6.7)	287.8 ± 169.3	NA
	PCS	64.9 ± 9.5	11 (39.3)	32.7 ± 5.1	11 (39.3)	8 (28.6)	4 (14.3)	296.5 ± 129.0	
Maloney 2019 [20]	ACS	NA	NA	30.3 *±* 3.7	6 (24.0)	5 (20.0)	NA	196.1 *±* 118.3	956.9 *±* 237
	PCS			33.1 *±* 6.1	9 (10.2)	20 (22.7)		198.1 *±* 122.6	956.9 *±* 237
Rodriguez 2021 [21]	ACS	64	641 (56.3)	29.7	324 (28.4)	268 (23.5)	227 (19.9)	187.7	785.9
	PCS	64	196 (57.1)	29.4	79 (23.0)	68 (19.8)	50 (14.6)	148.7	556.3
Toma 2023 [22]	ACS	59.13 *±* 11.21	65 (64.4)	30.36 *±* 5.6	6 (5.9)	16 (15.8)	7 (6.9)	130	NA
	PCS	63 *±* 6.78	56 (55.4)	31.7 *±* 4.2	25 (24.8)	48 (47.5)	18 (17.8)	263	
Demetrashvili 2025 [23]	ACS	57.4 ± 13.5	9 (40.9)	31.7 ± 6.8	9 (40.9)	2 (9.1)	2 (9.1)	235.2 ± 75.1	1002 ± 138.2
	PCS	58.7 ± 12.4	7 (33.3)	32.4 ± 7.3	7 (33.3)	2 (9.5)	3 (14.3)	241.7 ± 69.7	965 ± 162.5

## Risk of bias assessment

**Fig. 2:**
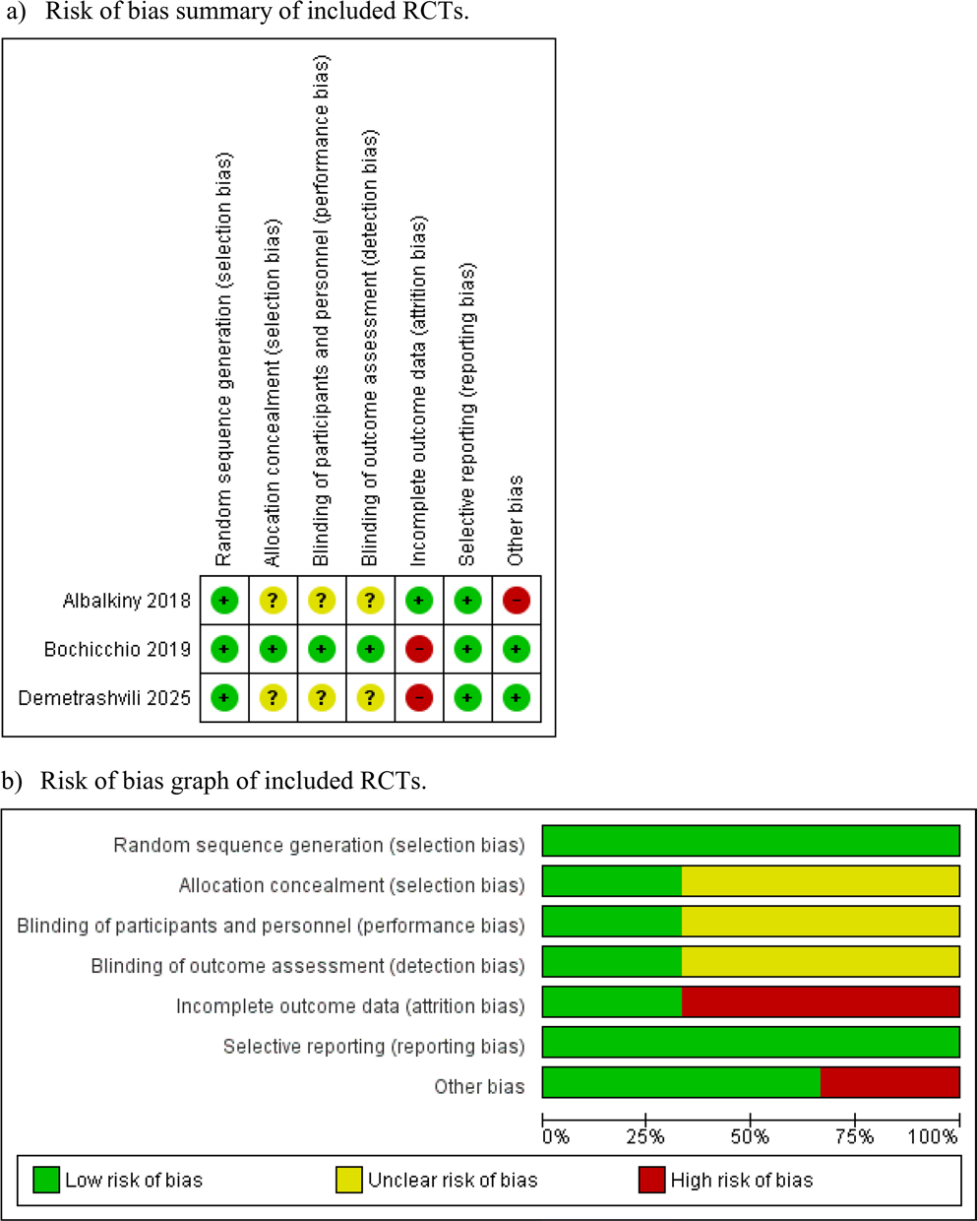
Forest plots of outcome measures. ACS, anterior component separation; PCS, posterior component separation with transversus abdominis muscle release. The solid squares denote the odds ratios (OR) or mean difference (MD). The horizontal lines represent the 95% confidence intervals (CIs), and the diamond denotes the pooled effect size. M-H, Mantel Haenszel test

Risk of bias assessment for RCTs and observational studies is illustrated in Fig. 2; Table 3, respectively. The three included RCTs generally demonstrated low risk of bias in most domains, with some unclear risk noted in blinding-related domains. For the five non-randomised studies, MINORS scores ranged from 15 to 19 (maximum 24), indicating overall moderate methodological quality. The lowest score was observed in the study by Toma et al. (2023) [22], primarily due to its retrospective design and lack of reporting on consecutive patients and sample size calculation. The highest scores were seen in the studies by Blair et al. [17], Maloney et al. [20], and Pereira Rodriguez et al. [21] (Table 4).

**Table 3:** Risk of bias assessment (observational studies) using the Newcastle-Ottawa scale

Study	Representativeness of the exposed cohort	Selection of the non-exposed cohort	Ascertainment of exposure	Demonstration that outcome of interest was not present at start of the study	Comparability of cohorts based on the design or analysis controlled for confounders	Assessment of outcome	Was follow-up long enough for outcomes to occur	Adequacy of follow-up of cohorts	Total
Karpata 2012 [16]	*	*	*	*		*	*	*	7
Blair 2016 [17]	*	*	*	*		*		*	6
Maloney 2019 [20]	*	*	*	*	*	*		*	7
Rodriguez 2021 [21]	*	*	*	*		*	*	*	7
Toma 2023 [22]	*	*	*	*	*	*	*	*	8

**Table 4:** Methodological index for non-randomised studies (MINORS) assessment

Study	Aim clearly stated (0–2)	Consecutive patients (0–2)	Prospective data collection (0–2)	Endpoints appropriate (0–2)	Unbiased endpoint assessment (0–2)	Adequate follow-up (0–2)	Loss to FU < 5% (0–2)	Sample size calculation (0–2)	Adequate control group (0–2)	Contemporary groups (0–2)	Baseline equivalence (0–2)	Adequate statistical analyses (0–2)	Total (/24)
Krpata 2012 [16]	2	2	0	2	1	1	0	0	2	2	2	2	16
Blair 2016 [17]	2	2	2	2	1	2	0	0	2	2	2	2	19
Maloney 2019 [20]	2	2	2	2	1	2	0	0	2	2	2	2	19
Pereira Rodriguez 2021 [21]	2	2	2	2	1	2	0	0	2	2	2	2	19
Toma 2023 [22]	2	1	0	2	1	1	0	0	2	2	2	2	15

*FU* follow-up

## Primary outcomes

### Postoperative wound complications

**Fig. 3:**
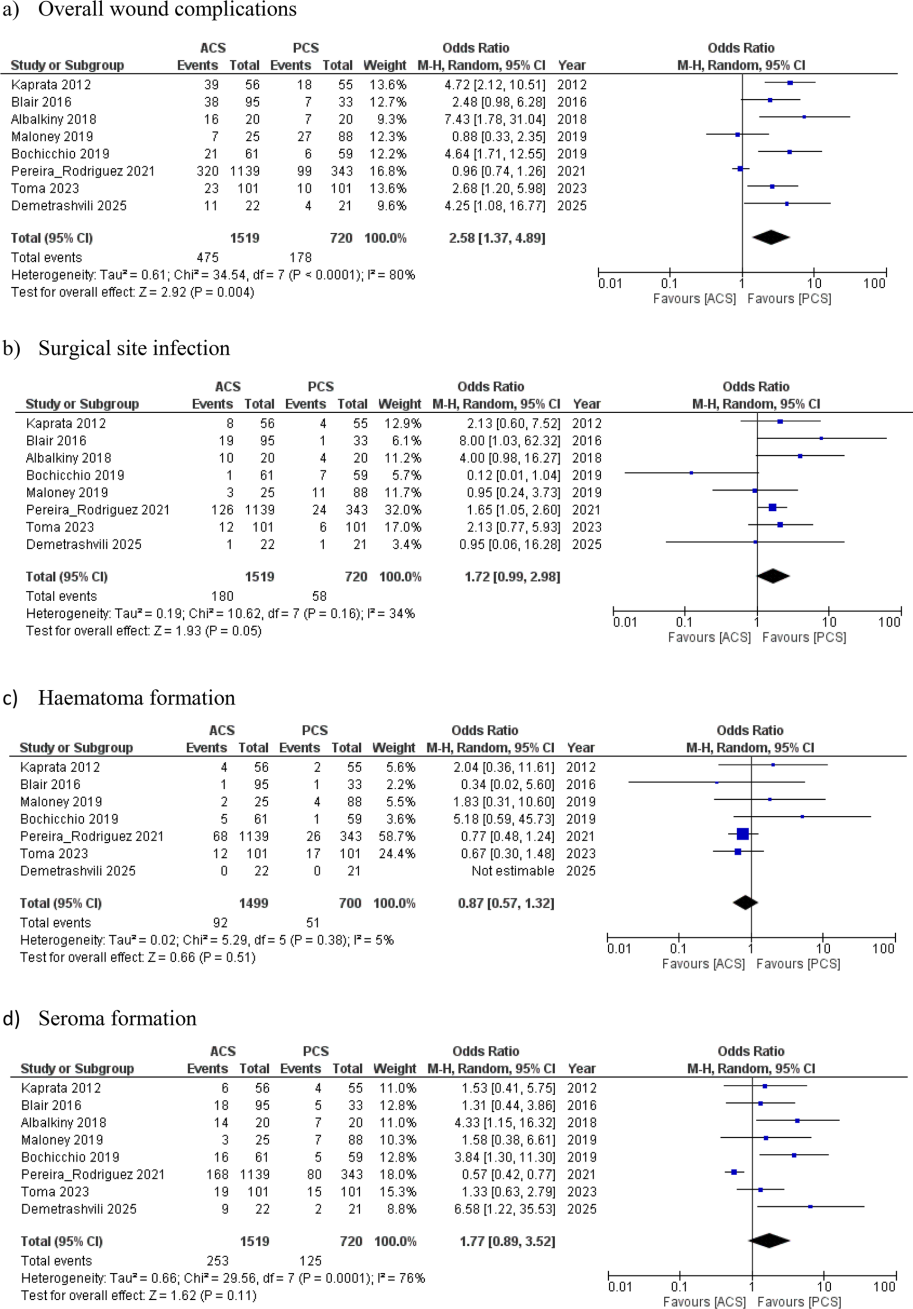

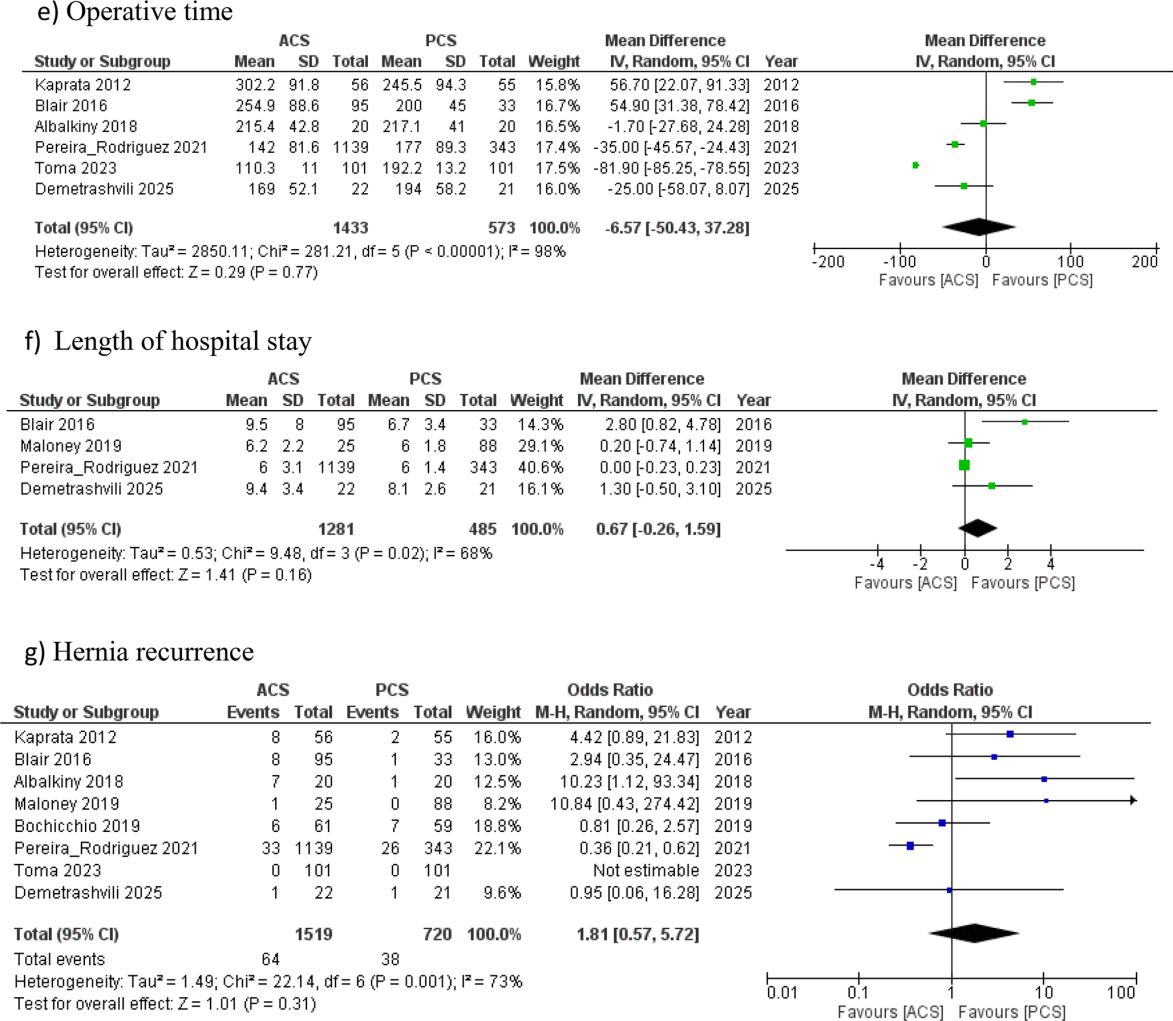
Forest plots of outcome measures. ACS, anterior component separation; PCS, posterior component separation with transversus abdominis muscle release. The solid squares denote the odds ratios (OR) or mean difference (MD). The horizontal lines represent the 95% confidence intervals (CIs), and the diamond denotes the pooled effect size. M-H, Mantel Haenszel test

The overall wound complications and SSI rates were reported in all eight studies (2,293 patients). PCSTAR group demonstrated a statistically significant lower risk for both overall wound complications and SSI compared with the ACS group; 24.7% vs. 31.3% for wound complications [OR 2.58, 95% CI (1.37, 4.89), *P* = 0.004] and 8.1% vs. 11.8% for SSI [OR 1.72, 95% CI (0.99, 2.98), *P* = 0.05] (Fig. 3). No significant differences were observed between the two groups for postoperative hematoma formation (reported in seven studies including 2,199 patients) [7.3% (PCSTAR) vs. 6.1% (ACS), OR 0.87, 95% CI (0.57, 1.32), *P* = 0.51] or seroma occurrence (reported in the all eight studies) [17.4% (PCSTAR) vs. 16.65% (ACS), OR 1.77, 95% CI (0.89, 3.52), *P* = 0.11] (Fig. 3).

## Secondary outcomes

### Operative time (minutes)

This outcome was reported in six studies with a total of 2,006 patients. Although the ACS group had a shorter operative duration, this difference was not statistically significant [MD −6.57, 95% CI [−50.43, 37.28], *P* = 0.77] (Fig. 3). Notably, there was a high level of heterogeneity among the included studies [I² = 98%, P = < 0.00001].

### Length of hospital stay (days)

LOS was reported in four studies with 1,766 patients. No statistically significant difference was observed between the two groups [8.1 ± 3.4 (PCSTAR) vs. 7.9 ± 3.11 (ACS), MD −0.67, 95% CI (−0.26,1.59), *P* = 0.16] (Fig. 3). Cochran Q test showed substantial heterogeneity among the included studies [I^2^ = 68%, *P* = 0.02].

### Recurrence rate

The hernia recurrence rate was reported in seven studies involving a total of 2,239 patients. Similar rates were observed between the two groups [5.3% (PCSTAR) vs. 4.2% (ACS), OR 1.81, 95% CI (0.57, 5.72), *P* = 0.031] (Fig. 3). There was a high level of heterogeneity among the included studies [I² = 73%, *P* = 0.001].

## Sensitivity analysis

No change in the direction of the estimated pooled effect size was detected when using RD or RR for dichotomous outcomes. Similarly, excluding one study at a time did not show significant discrepancies or deviation from our initial results.

## Discussion

We aimed to compare two surgical approaches (ACS & PCSTAR) in the management of large VHs. To our knowledge, this is the largest dataset (2,293 patients) available that provides a direct comparison with the exclusion of single-arm studies (done to prevent discrepancies in results due to mismatched patient characteristics).

The repair of large VHs remains challenging, and several prevention techniques, such as prophylactic mesh closure, have been described [24, 25]. This study included patients with an abdominal wall defect >10 cm (width and/or length). To repair such sizeable defects, CST was introduced and subsequently modified [26]. The principles of CST enable tissue dissection and approximation of fascial layers, and combined with the release of the transversus abdominis muscle, help create a space for mesh placement, providing further support and augmentation in VH repair. Several approaches to CS have been described in the literature [27], including ACS and PCSTAR. However, there is ongoing debate and controversy surrounding the outcomes of these repairs, including the impact on quality-of-life metrics.

CST is associated with a high risk of developing post-operative wound issues [28, 29]. In the present study, overall wound morbidity was 31.3% in the ACS group and 24.7% in the PCSTAR group. This difference was statistically significant, favouring the PCSTAR technique and in agreement with the findings of Oprea et al. [8]. They reported lower overall wound morbidity in patients undergoing PCSTAR compared with ACS repair (28.5%). This was also reported in a retrospective study [30], which identified 35 events of wound complications in 30 ACS patients. Lower wound complication rates utilising the PCSTAR technique have also been highlighted by Maloney et al. [31].

This study found no significant difference in hematoma and seroma formation between the two groups. Seroma formation occurs in approximately 5% to 20% of patients undergoing hernia repair (dependent on surgical approach) [32]. The placement of mesh and extensive tissue dissection have been suggested as possible causes for this phenomenon. The use of surgical drains may prevent fluid accumulation in the early postoperative period; however, the longer-term benefits are unclear [33]. For secondary outcomes, both groups showed comparable results regarding total operative time, LOS, and hernia recurrence rates.

However, lower rates of SSI and overall wound complications were observed with the PCSTAR technique, contributing to more favourable patient outcomes. This is important as patients with VHs often have other co-morbidities (obesity, cardio-respiratory conditions). Therefore, surgical technique and its sequela can have detrimental and considerable effects on quality of life.

Cadaveric studies are available showing the length of fascial advancement achieved by these surgical approaches [34–36]. For instance, Loh et al. [34] compared cadaveric ACS vs. PCSTAR in ten cadavers with various ventral hernia defect sizes. They showed that ACS, on average, provided more medial advancement (35 mm) than PCSTAR (24 mm). This difference is thought to be related to the insertion of the external oblique muscle, providing a tethering effect compared to TAR, which inserts into the rectus sheath. Moreover, the dissecting plane between the external and internal oblique muscles allows more medial advancement of the rectus block compared with TAR.

However, data from a second cadaveric study [35] showed conflicting results with an average advancement with ACS of 3.38 cm (+/- 0.69), PCS alone of 3.98 cm (+/- 0.94), increasing to 4.31 cm (+/- 0.89) with the addition of TAR (PCSTAR). A further study [34] supported the addition of TAR release with the CS technique, increasing the advancement of the anterior rectus sheath by 102% and the posterior rectus sheath by 129% from baseline. These results may not necessarily translate into ‘live’ patient scenarios due to factors such as tissue elasticity, size, location of the abdominal wall defect, and abdominal wall compliance. Consequently, the absence of such data on the degree of fascial length achieved by the various surgical techniques makes it difficult to make robust recommendations and draw conclusions. Future, well-designed RCTs are necessary to compare these techniques directly, incorporating intra-operative measurements of tissue advancement as a key parameter. Furthermore, they can address the heterogeneity originating from differences in patient selection and provide clearer guidance on technique selection based on the size of the hernial defect.

Another challenge for patients presenting with large VHs is often the presence of co-morbidities that can prolong post-operative recovery and complicate preoperative assessment. These include cardio-respiratory conditions, diabetes, morbid obesity, or malnutrition (Table 2).

Delays in surgical repair may lead to further enlargement of the abdominal viscera, increasing the size of the hernial defect and causing loss of domain [36]. This added complexity can make the surgical procedure even more challenging and the post-operative recovery more difficult. Preoperative measures, such as Botox injection and pneumoperitoneum, have been proposed to expand the abdominal cavity, allowing for more medial advancement of the fascial layers [37, 38].

In the present study, the recurrence rates were 4.2% in the ACS group and 5.3% in the PCSTAR group (*P* = 0.31). Although these rates are encouraging, they should be interpreted with caution due to the short follow-up period in some included studies [22].

The use of mesh augmentation in abdominal wall reconstruction (AWR) helps to reduce recurrence [39]. However, there is debate over the type of mesh, mesh size, and area of coverage. Different mesh sizes were used in our included studies (Table 2), and the mesh-to-defect ratio remains an important consideration when selecting mesh type. Mesh positioning and fixation sites are other challenges encountered during these repairs [40].

This study is not without its limitations. Firstly, the nature of the included studies introduces considerable bias and may contribute to increased heterogeneity. Another drawback is the limited data available on the length of fascial advancement achieved through retro-fascial dissection or TAR release, which could help towards a more consistent approach based on defect size. Additionally, short follow-up periods in some studies may impact the validity and generalisability of outcomes, such as recurrence rates.

Moreover, only three RCTs were included in our analysis, all of which carried a moderate risk of attrition bias. The lack of standardised surgical techniques and differences in surgical approaches between centres likely introduces heterogeneity and influences outcomes, further emphasising the need for future well-designed RCTs.

Two included studies had specific design features that could introduce between-study heterogeneity. Toma et al. [22] included both primary and recurrent ventral hernias and had a relatively short follow-up period (3 months). This may underestimate the actual recurrence rate, and has to be considered when interpreting our findings. Additionally, Maloney et al. [20] included a proportion of patients with both flank and ventral hernias. Although sensitivity analyses (conducted through excluding these studies) showed no meaningful change in the direction of pooled outcomes, these design elements may well influence effect estimates and again need to be considered when interpreting study results.

Lastly, this study did not address patient quality of life parameters and metrics, necessitating the need for long-term follow-up and patient-led surveys to understand post-operative issues and challenges.

## Conclusion

CST for the repair of IHs is effective, with ACS and PCSTAR being the two most popular choices. Both techniques show comparable post-operative outcomes. PCSTAR seems to be safer in reducing overall wound complications and SSIs. However, these findings should be interpreted with caution, given the limited number of randomised trials and the variation in surgical technique across studies. The lack of real patient data on actual fascial length achieved by the various techniques is a limiting factor in drawing robust conclusions and making recommendations. Adequately powered, well-designed RCTs are needed to bridge this knowledge gap, determine outcomes directly between ACS and PCSTAR, and help standardise surgical technique. These will be useful in aiding clinical decision-making and lead to improved patient outcomes.

## Supplementary Information

Below is the link to the electronic supplementary material.

Supplementary Material 1

Supplementary Material 2

## Data Availability

The authors confirm that the data supporting the findings of this study are available within the article and the supplementary material. The raw data are available from the corresponding author on request.
