# Supplementary material for: Outcomes of anterior component separation versus posterior component separation with transversus abdominis muscle release for large incisional hernias: a systematic review and meta-analysis
Source: Hernia. 2025 Oct 9;29(1):289. doi: 10.1007/s10029-025-03487-5 (PMC12511176; doi:10.1007/s10029-025-03487-5)
Supplement: Supplementary file 1 — Supplementary Material 1 [file 10029_2025_3487_MOESM1_ESM.docx]

30/08/2025

Dear Editor,

Many thanks for considering our manuscript entitled "Outcomes of anterior component separation versus posterior component separation with transversus abdominis muscle release for large incisional hernias: a systematic review and meta-analysis" (Ref: Ms. No. HERN-D-25-00468) for revision. Our evidence synthesis group is grateful to you and the reviewers for their valid comments, which undoubtedly will enhance the quality of our manuscript.

Please find below our responses to the reviewers' comments.

Kind regards,

The Authorship team

**Author’s response to reviewers’ comments**

**Reviewer#1:**

Dear authors,
It was a pleasure to read your manuscript, " Outcomes of anterior component separation versus posterior component separation with transversus abdominis muscle release for large ventral hernias: A systematic review and meta-analysis". Although several reviews on the topic have been published in recent years, I appreciate the boldness of your approach in comparing ACS vs PCSTAR only from double-armed studies. We need more accurate data about these techniques to improve outcomes and quality of life for our patients. I had high expectations after reading the abstract, but the final result fell short of my expectations. There are several concerns to be addressed:

**Comment 1.** I do not understand why you prefer to use the term "ventral hernia" instead of "incisional hernia"? There are different pathologies with different etiologies ( as you well describe in the background section), different approaches ( rarely a ventral hernia reaches large defects that require component separation techniques), and most importantly, with different outcomes. Moreover, all the papers included in your meta-analysis refer to incisional hernias, not to ventral. So, it is a must to revise the title according to the topic and references. Using the term ventral hernia is confusing and unrealistic.

**Response 1:** Thank you for taking the time to review our manuscript providing valuable feedback and highlighting this point. We agree that ventral hernia is a broader term. Therefore, based on your suggestion we have now revised the title to read ‘Outcomes of anterior component separation versus posterior component separation with transversus abdominis muscle release for large incisional hernias: a systematic review and meta-analysis’.

The necessary changes have also been made in the body of the manuscript, where applicable.

**Comment 2.** Both abstract and plain text need to contain the specific interval of time of the literature search ( eg, from January 1899 to April 2030 ) because, as you state, the goal is an update analysis.

**Response**: Thank you for this comment. We have now clearly specified the search period as “from January 1990 to June 2025” in both the abstract and methods section.

**Abstract → Methods**

*(published between January 1990 - June 2025)*

**Methods → study selection**

*and published between January 1990 - June 2025*

**Comment 3.** In the background section you described the principles of the component separation but your description is somehow confusing and the details are for the ACS. If so, please revise and specify

**Response**: Thank you for pointing this out. We have now amended the introduction to take this comment into account and clarify differences between ACS and PCSTAR.

**Introduction → paragraph 3**

*The component separation technique (CST) developed and popularised in the early 1990s allows large abdominal wall defects to be repaired without the need for bridging mesh. [4] It achieves this through the medial advancement of abdominal wall muscles via the release of surrounding tissue planes, favouring fascial closure. [5] In anterior component separation (ACS), dissection is performed in the subcutaneous plane, and the external oblique aponeurosis incised lateral to the rectus sheath facilitating closure of the hernial defect. [6]* ***In posterior component separation with transversus abdominis release (PCSTAR)*** *a more recent modification - the retro-rectus space is developed and the transversus abdominis muscle released at its insertion. This creates a large, well-vascularised space for mesh placement, often avoiding the need for subcutaneous dissection. The technique allows closure of large midline defects with reduced risk of wound morbidity compared with ACS, especially in obese and multi-morbid patients.* *[7]*

**Comment 4.** please describe the method used for quality of studies evaluation. MINOR's criteria is a good descriptor (Slim K, Nini E, Forestier D et al (2003) Methodological index for non-randomized studies (minors): development and validation of a new instrument. ANZ J Surg 73:712-**716.** [https://gbr01.safelinks.protection.outlook.com/?url=https%3A%2F%2Fdoi.org%2F10.1046%2Fj.1445-2197.2003.02748.x&data=05%7C02%7Cshafquatzaman%40nhs.net%7C3eb8a845f7ea4eb1010c08ddc83b0ecb%7C37c354b285b047f5b22207b48d774ee3%7C0%7C0%7C638886878149033858%7CUnknown%7CTWFpbGZsb3d8eyJFbXB0eU1hcGkiOnRydWUsIlYiOiIwLjAuMDAwMCIsIlAiOiJXaW4zMiIsIkFOIjoiTWFpbCIsIldUIjoyfQ%3D%3D%7C0%7C%7C%7C&sdata=VbkHuIg0JKBd%2FHKDi271tm%2BhmeZHDViihKITYuXMGqc%3D&reserved=0)](https://gbr01.safelinks.protection.outlook.com/?url=https%3A%2F%2Fdoi.org%2F10.1046%2Fj.1445-2197.2003.02748.x&data=05%7C02%7Cm.issa2%40nhs.net%7C3a46d5e395a74786c63c08ddc840f758%7C37c354b285b047f5b22207b48d774ee3%7C0%7C0%7C638886904056013447%7CUnknown%7CTWFpbGZsb3d8eyJFbXB0eU1hcGkiOnRydWUsIlYiOiIwLjAuMDAwMCIsIlAiOiJXaW4zMiIsIkFOIjoiTWFpbCIsIldUIjoyfQ%3D%3D%7C0%7C%7C%7C&sdata=knyqoK7jmfjtYSjjNnqBh1pjQUuV3kmRbBAwcDKpeW4%3D&reserved=0). this must be found also in the results. You can add a new column to the table describing the main characteristics of the eincluded studies.

**Response**: Thank you for this valuable suggestion. We have now clarified our methodology for assessing the quality of included studies. For RCTs, we used the Cochrane Risk of Bias tool, and for non-randomised studies, we applied the Methodological Index for Non-Randomised Studies (MINORS) criteria as recommended (Slim et al., 2003). This has been described in the Methods section under “Risk of Bias Assessment.” In the Results section, we report the findings from both tools, including a new dedicated table (Table 4) that presents the MINORS scores for each non-randomised study. The figure for the RCT risk of bias assessment (Figure 2) has also been updated to include all relevant trials. Also, we incorporated this information into the abstract, methods, and results sections. Please note that we identified Albalkiny 2018 as an RCT.

**Methods → Risk of bias assessment**

*For non-randomised studies, methodological quality was assessed using the Methodological Index for Non-Randomised Studies (MINORS) tool [14]. This validated scoring system includes 12 items, each scored from 0 to 2, with a maximum possible score of 24 for comparative studies. Two reviewers independently scored each study, and any disagreements were resolved through discussion and reaching a consensus.*

**Results → Risk of bias assessment**

*For the five non-randomised studies, MINORS scores ranged from 15 to 19 (maximum 24), indicating overall moderate methodological quality. The lowest score was observed in the study by Toma et al. (2023) [22], primarily due to its retrospective design and lack of reporting on consecutive patients and sample size calculation. The highest scores were seen in the studies by Blair et al. [17], Maloney et al. [20], and Pereira Rodriguez et al. [21] (Table 4).*

**Comment 5.** please define outcomes according to the literature (. National Healthcare Safety Network (2022) Surgical Site Infection Event (SSI). [https://gbr01.safelinks.protection.outlook.com/?url=http%3A%2F%2Fcdc.gov%2Fnhsn%2Fpdfs%2Fpscmanual%2F9pscssicurrent.pdf&data=05%7C02%7Cshafquatzaman%40nhs.net%7C3eb8a845f7ea4eb1010c08ddc83b0ecb%7C37c354b285b047f5b22207b48d774ee3%7C0%7C0%7C638886878149060398%7CUnknown%7CTWFpbGZsb3d8eyJFbXB0eU1hcGkiOnRydWUsIlYiOiIwLjAuMDAwMCIsIlAiOiJXaW4zMiIsIkFOIjoiTWFpbCIsIldUIjoyfQ%3D%3D%7C0%7C%7C%7C&sdata=52hroj2CF0Clw8Po0GujpW9Fnd4plagqUB%2FHWOqOcP0%3D&reserved=0](https://gbr01.safelinks.protection.outlook.com/?url=http%3A%2F%2Fcdc.gov%2Fnhsn%2Fpdfs%2Fpscmanual%2F9pscssicurrent.pdf&data=05%7C02%7Cm.issa2%40nhs.net%7C3a46d5e395a74786c63c08ddc840f758%7C37c354b285b047f5b22207b48d774ee3%7C0%7C0%7C638886904056028082%7CUnknown%7CTWFpbGZsb3d8eyJFbXB0eU1hcGkiOnRydWUsIlYiOiIwLjAuMDAwMCIsIlAiOiJXaW4zMiIsIkFOIjoiTWFpbCIsIldUIjoyfQ%3D%3D%7C0%7C%7C%7C&sdata=QhMsdwvi77s%2BVHKyqOvwShIofLQjwfzjiHhS5KAwtXU%3D&reserved=0). Accessed 19 Feb 2022)

**Response:** Thank you for highlighting this point. We have revised our definition of surgical site infections in accordance with the 2022 CDC NHSN criteria. The definition is now included in the Methods section, and the source has been cited accordingly in the References.

**Methods → study outcomes**

*SSIs were defined according to the National Healthcare Safety Network (NHSN, CDC 2022) criteria, which categorises SSIs into superficial incisional, deep incisional, and organ/space infections occurring within 30 days of the procedure (or within 90 days if prosthetic material is implanted), and meeting specific clinical, laboratory, or radiological findings. [12]*

**References → Reference 12**

12.	Centres for Disease Control and Prevention (CDC). National Healthcare Safety Network (NHSN) Patient Safety Component Manual: Surgical Site Infection (SSI) Event. Atlanta, GA: Centres for Disease Control and Prevention; 2022. Available at: http://cdc.gov/nhsn/pdfs/pscmanual/9pscssicurrent.pdf. Accessed 7 Aug 2025.

**Comment 6.** Section Results needs major revision. In fact is the mirror of your research and needs to be extended: add a PRISMA flow chart according to the new agreements ([https://gbr01.safelinks.protection.outlook.com/?url=https%3A%2F%2Fguides.lib.unc.edu%2Fprisma&data=05%7C02%7Cshafquatzaman%40nhs.net%7C3eb8a845f7ea4eb1010c08ddc83b0ecb%7C37c354b285b047f5b22207b48d774ee3%7C0%7C0%7C638886878149083688%7CUnknown%7CTWFpbGZsb3d8eyJFbXB0eU1hcGkiOnRydWUsIlYiOiIwLjAuMDAwMCIsIlAiOiJXaW4zMiIsIkFOIjoiTWFpbCIsIldUIjoyfQ%3D%3D%7C0%7C%7C%7C&sdata=ZqeUO6MbmKL7aPrIunuBzEyL7zoHrTqhEtvdzYIjWdo%3D&reserved=0](https://gbr01.safelinks.protection.outlook.com/?url=https%3A%2F%2Fguides.lib.unc.edu%2Fprisma&data=05%7C02%7Cm.issa2%40nhs.net%7C3a46d5e395a74786c63c08ddc840f758%7C37c354b285b047f5b22207b48d774ee3%7C0%7C0%7C638886904056045820%7CUnknown%7CTWFpbGZsb3d8eyJFbXB0eU1hcGkiOnRydWUsIlYiOiIwLjAuMDAwMCIsIlAiOiJXaW4zMiIsIkFOIjoiTWFpbCIsIldUIjoyfQ%3D%3D%7C0%7C%7C%7C&sdata=cGRXROuox9ZMqezMdZCVAbTjua7NdV1x20%2BeR4WqSHk%3D&reserved=0)); insert in the results pooled results for demographics ( number of patients included, male to female ratio, age, BMI etc), intraoperative characteristics ( defect dimensions, area, operative time, type of mesh, fascia closure). All of these cofounders are important factors predicting complications and are directly related to them. So, please improve the results. Compare all these things and it will be better.

**Response**: Thank you for this invaluable advice.

Figure 1 has now been updated according to the 2020 PRISMA flow chart.

For the included patient characteristics, this is a valid suggestion that will enhance our results and make them more meaningful. We have updated the results section by adding details on patient characteristics and baseline demographics; expanded table 2 - showing pooled number of confounders (gender, smoking status, age, BMI, COPD) and size of hernial defect and mesh used for repair.

**Results → Patient Characteristics and Baseline Demographics**

*A total of 2,293 patients were included in this analysis (1,573 in ACS group and 720 patients in PCSTAR group). The ACS group consisted of 842 males (53.5%) and 731 females (46.5%), whilst the PCSTAR group had 378 males (52.5%) and 342 females (47.5%), demonstrating comparable gender distribution between the two groups. Notably, one study included a proportion of patients with flank hernias in addition to ventral hernias [20], and two studies included both primary and recurrent ventral hernias [20,22]. The average age of our cohort was 57.5 years (ACS group) and 58.6 years (PCSTAR group), respectively. Mean body mass index (BMI) was also similar across the two groups (32.7 kg/m² for ACS and 32.5 kg/m² for PCSTAR), indicating Class 1 obesity.*

*For co-morbidities, 18.8% of patients in the ACS group and 36.4% in the PCSTAR group were either current or former smokers. Diabetes mellitus was reported in 21.6% of ACS and 28.2% of PCSTAR patients. COPD was present in 15.3% of ACS and 15.7% of PCSTAR patients, reflecting a similar pulmonary risk profile between the two groups. The demographics and operative profiles of the included patients are summarised in Table 2.*

**Comment 7.** please complete the evaluation of the risk of bias for all included studies. You have enough statistical instruments in Revman.

**Response**: Thank you for this comment.

With the addition of the MINORS assessment, all eight included studies have now been evaluated for methodological quality using validated tools appropriate to study design.

**comment 8.** please insert your Compliance with Ethical Standards ( see Instructions for the authors)

**Response**: Thank you.

A statement on compliance with ethical standards has now been included following the conclusion section.

**Reviewer#2:**

This paper focuses on the outcome of anterior versus posterior component separation (TAR) in a review with meta-analysis. This update of the existing literature is of practical value for the evaluation of the two methods in terms of efficiency, outcome and occurrence of complications.
 A few points of criticism and comments on this manuscript may be permitted:

**Comment 1.** why was this review with meta-analysis not registered in advance, as is customary (e.g. PROSPERO)?

**Response**: Thank you for taking the time to review our manuscript and providing feedback.

We agree that prospective PROSPERO registration is important and good practice for openness and transparency in research. On this occasion, regrettably this was a complete oversight on the part of the authorship team and will definitely be considered for future publications. We have acknowledged this point in our methods section (study design paragraph).

**Methods → Study design**

*Although the study was not prospectively registered on a systematic review registry such as PROSPERO [11], it was conducted in accordance with PRISMA guidelines and adhered to Cochrane methodology to ensure transparency and quality.*

**Comment 2.** when assessing the risk of bias, it is noticeable that the study by Toma et al was included here with a follow-up of only 3 months. in addition, primary and recurrent hernias of the abdominal wall were included in the study, which is not acceptable due to the different genesis and the resulting non-comparability. therefore, this study should be excluded from the meta-analysis, especially with regard to the occurrence of recurrence (Newcastle-Ottawa Scale in relation to f.u.!!).

**Response:** We appreciate the reviewer’s comment and observation. We acknowledge that Toma et al. included both primary and recurrent ventral hernias and had a relatively short follow-up period of 3 months. We included this study because it offers recent comparative data relevant to the surgical techniques of interest. To address potential bias, we have already performed a sensitivity analysis (leave-one) excluding Toma et al., which showed no significant change in the pooled effect estimates. These results are presented in the Results section, and the short follow-up and mixed hernia types have been acknowledged as limitations in the revised Discussion section.

**Discussion → Limitations**

*Two included studies had specific design features that could introduce between-study heterogeneity. Toma et al. [22] included both primary and recurrent ventral hernias and had a relatively short follow-up period (3 months). This may underestimate the actual recurrence rate, and has to be considered when interpreting our findings.*

**Results → sensitivity analysis**

*No change in the direction of the estimated pooled effect size was detected when using RD or RR for dichotomous outcomes. Similarly, excluding one study at a time did not show significant discrepancies or deviation from our initial results.*

**Comment 3.** a similar problem arises in the study by Maloney et al. where flank hernias were also included.

**Response:** We thank the reviewer for highlighting this point and acknowledge their concern.

Maloney et al. included a proportion of patients with flank hernias alongside those with ventral hernias. This study was retained because it contains relevant comparative data for the surgical techniques under investigation. We conducted a sensitivity (leave-one) analysis excluding this study, which did not alter the pooled effect estimates. The inclusion of flank hernias and their potential to introduce heterogeneity has been clearly acknowledged in the revised Discussion section.

**Discussion → Limitations**

*Additionally, Maloney et al. [20] included a proportion of patients with both flank and ventral hernias. Although sensitivity analyses (conducted through excluding these studies) showed no meaningful change in the direction of pooled outcomes, these design elements may well influence effect estimates and again need to be considered when interpreting study results.*

**Results → sensitivity analysis**

*No change in the direction of the estimated pooled effect size was detected when using RD or RR for dichotomous outcomes. Similarly, excluding one study at a time did not show significant discrepancies from our original results.*

**Comment 4.** the final conclusion of this review is very limited with the inclusion of only 2 RCTs, both of which have a high risk of attrition. In addition, the interpretation of the data seems to be a major weakness due to the lack of standardization of the technique in the studies.

**Response**: Thank you for this comment.

We have revised both the abstract and the conclusion, and a statement has been added to the limitations in the final paragraph to acknowledge this concern.

**Abstract → conclusion**

*A small number of included RCTs mandate that* further adequately powered, well-designed RCTs are required to validate these findings.

**Discussion → conclusion**

*Conclusion: CST for the repair of IHs is effective, with ACS and PCSTAR being the two most popular choices. Both techniques show comparable post-operative outcomes. PCSTAR seems to be safer in reducing overall wound complications and SSIs. However, these findings should be interpreted with caution, given the limited number of randomised trials and the variation in surgical technique across studies. The lack of real patient data on actual fascial length achieved by the various techniques is a limiting factor in drawing robust conclusions and making recommendations. Adequately powered, well-designed RCTs are needed to bridge this knowledge gap, determine outcomes directly between ACS and PCSTAR, and help standardise surgical technique. These will be useful in aiding clinical decision-making and lead to improved patient outcomes.*
